# Metastasis of Clear Cell Renal Cell Carcinoma to the Parotid Gland: A Case Report

**DOI:** 10.7759/cureus.43676

**Published:** 2023-08-17

**Authors:** Mahdi Aljawad, Mohammed K Alharbi, Samar M Algahtani, Hanan M Mughallis, Shaykhah M Almhna

**Affiliations:** 1 Radiology, Qatif Central Hospital, Al Qatif, SAU; 2 General Practice, Al-Rayan Colleges, Medina, SAU; 3 General Practice, King Saud Bin Abdulaziz University for Health Sciences, Riyadh, SAU; 4 General Practice, Ibn Sina National College For Medical Studies, Jeddah, SAU; 5 General Practice, Al Jouf University, Al-Jouf, SAU

**Keywords:** surgical case report, head and neck tumors, magnetic resonance imaging, clear cell renal carcinoma, parotid gland metastasis

## Abstract

Parotid gland metastases from distant primary malignancies are uncommon and present diagnostic challenges for clinicians. We present the case of a 65-year-old male with a history of clear cell subtype of renal cell carcinoma who presented with a painless swelling in the right parotid region. His medical history was significant for a right-sided renal cell carcinoma, for which he had undergone a radical nephrectomy five years ago. The patient's physical examination revealed a firm, non-tender mass in the right parotid gland region. Imaging studies, including ultrasound and contrast-enhanced computed tomography, confirmed the presence of a solid-enhancing lesion within the parotid gland. Fine-needle aspiration biopsy provided histological evidence of malignant cells with features consistent with a clear renal cell carcinoma cell subtype. This is consistent with diagnosing metastatic renal cell carcinoma to the parotid gland. This case highlights the significance of considering metastatic disease in the differential diagnosis of parotid swellings, particularly in patients with a history of remote malignancy. Systemic targeted therapy, with a tyrosine kinase inhibitor, emerged as an effective treatment option, emphasizing the importance of personalized approaches in managing rare clinical scenarios. Tailored management is crucial in optimizing outcomes for patients with parotid gland metastases, improving their overall prognosis and quality of life.

## Introduction

Renal cell carcinoma is a common malignancy of the urinary system, with a propensity for metastatic spread to various organs. The most common sites of metastasis include the lungs, bones, liver, and regional lymph nodes. However, metastasis to the parotid gland is an uncommon and intriguing clinical entity. Parotid gland metastases are rare, representing less than 5% of all malignant tumors affecting the salivary glands [1]. The parotid gland is the largest salivary gland, and its involvement in metastatic disease is predominantly associated with primary cutaneous malignancies.

Additionally, most patients present with multiple metastases, and solitary parotid location is extremely rare. When the primary cancer originates in sites below the clavicle, metastases to the head and neck are uncommon. Furthermore, metastasis to the parotid gland rarely occurs and usually proceeds from skin cancers of the head and neck, such as melanoma in 45% of cases and squamous cell carcinoma in 37% [1-2]. The mechanism by which a renal cell carcinoma reaches the parotid gland is likely through hematogenous spread. Renal cell carcinomas are hypervascular tumors associated with multiple arteriovenous shunts. Since kidneys receive 25% of the circulating blood volume, renal cell carcinoma has a high potential for hematogenous spreading. This report presents a compelling case of a 65-year-old male with a remote history of renal cell carcinoma who presented with parotid swelling.

## Case presentation

A 65-year-old male presented with a painless swelling in the right parotid region that had gradually increased in size over the past six months. The patient reported no associated symptoms such as pain, fever, weight loss, or changes in facial sensation. His medical history was significant for a right-sided renal cell carcinoma, for which he had undergone a radical nephrectomy five years earlier. The histopathological examination at that time had revealed a clear cell subtype of renal cell carcinoma, and the patient had remained disease-free since then, with regular follow-up visits.

Upon physical examination, a firm and non-tender mass approximately 4 cm in diameter was palpable in the right parotid gland region. The overlying skin appeared normal, with no signs of inflammation or local infection. The facial nerve function was intact, and no other significant abnormalities were noted on cranial nerve examination. The rest of the head and neck examination was unremarkable. Given the patient's history of renal cell carcinoma, a metastatic lesion was suspected as a possible etiology for parotid swelling. To confirm the diagnosis, a comprehensive work-up was initiated. Initial laboratory investigations were within normal limits, including complete blood count, renal function tests, liver function tests, and electrolyte levels. However, imaging studies were deemed necessary for further evaluation.

**Figure 1:**
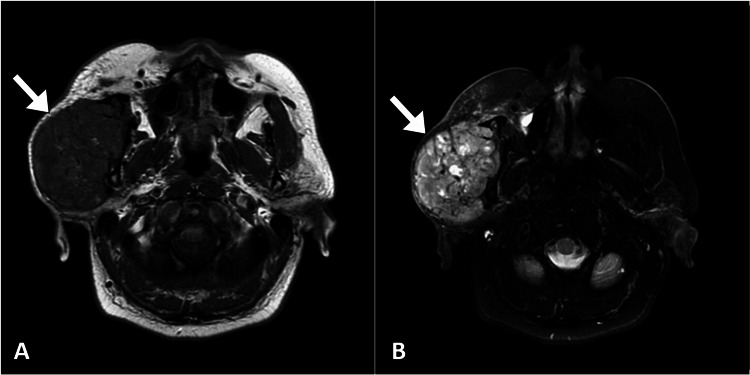
Axial T1-weighted (A) and T2-weighted (B) MR images show a heterogeneous mass (arrow) in the right parotid gland. MR: magnetic resonance

Ultrasound examination of the parotid region revealed a well-defined hypoechoic lesion with internal vascularity, measuring approximately 4.5x 3.8x3.2 cm, located within the superficial lobe of the right parotid gland. The contralateral parotid gland and the salivary ducts appeared normal on ultrasound. Additionally, a magnetic resonance imaging of the head was performed, which confirmed the presence of a solid enhancing lesion in the right parotid gland, consistent with a neoplastic process. The lesion showed no evidence of invasion into adjacent structures, and no other distant metastases were detected (Figure 1).

**Figure 2:**
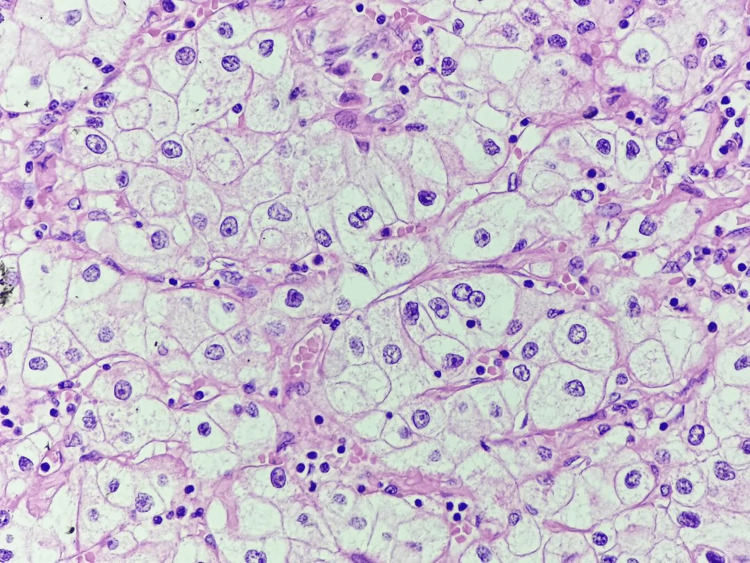
Microscopic examination reveals clusters of malignant cells with clear cytoplasm, confirming the diagnosis of metastatic renal cell carcinoma.

Based on the imaging findings and the patient's medical history, the differential diagnosis included various benign and malignant parotid gland tumors and metastasis from his previously treated renal cell carcinoma. A biopsy was performed to obtain a tissue sample for histopathological examination. The histopathological examination revealed clusters of malignant cells with abundant clear cytoplasm, consistent with metastatic clear cell subtype of renal cell carcinoma (Figure 2). Given the correlation between the patient's history of renal cell carcinoma and the histopathological findings, a diagnosis of metastatic clear cell subtype of renal cell carcinoma to the right parotid gland was established.

The patient's case was discussed in a multidisciplinary tumor board meeting, and it was decided to avoid further surgical intervention per the patient's preference. Hence, the patient was recommended for systemic targeted therapy with tyrosine kinase inhibitors, a treatment option that has shown promising results in advanced renal cell carcinoma cases. During his hospital course, the patient was started on the recommended targeted therapy, and regular follow-up visits were scheduled to monitor treatment response and manage any adverse effects.

## Discussion

Diagnosing metastatic clear cell subtype of renal cell carcinoma to the parotid gland in our patient raises several intriguing clinical and diagnostic considerations. Parotid gland metastases are rare, and their occurrence from distant primary sites, such as renal tumors, is even rarer [1-2]. This discussion will delve into various aspects of the case, compare our findings with the existing literature, and explore the implications for clinical practice.

Firstly, the clinical presentation of parotid swelling in our patient with a remote history of renal cell carcinoma posed a diagnostic challenge. The initial differential diagnosis included benign and malignant etiologies, such as pleomorphic adenoma, Warthin tumor, mucoepidermoid carcinoma, and acinic cell carcinoma, which are more common primary parotid gland tumors [3]. Nonetheless, the patient's medical history of renal cell carcinoma served as a critical clue in considering metastasis as a potential cause of parotid swelling. This highlights the significance of thorough history-taking and its impact on guiding subsequent investigations.

The imaging studies played a pivotal role in confirming the presence of a solid-enhancing lesion within the right parotid gland. The findings were consistent with a neoplastic process, and the lesion demonstrated characteristics similar to the primary renal cell carcinoma identified during the patient's prior nephrectomy. Using imaging modalities, such as ultrasound and magnetic resonance imaging, facilitated the localization and characterization of the parotid mass [2-3]. Biopsy remains a valuable diagnostic tool in evaluating parotid swellings. In our case, biopsy provided histological evidence of malignant cells with abundant clear cytoplasm resembling the histological features of the clear cell subtype of renal cell carcinoma. The combination of imaging and histological findings strengthened the suspicion of metastatic renal cell carcinoma to the parotid gland.

The management of parotid gland metastases presents a challenging scenario for clinicians. Surgical excision, traditionally considered the mainstay of treatment for primary parotid malignancies, may not be the optimal approach in cases of metastatic disease. The location of the parotid gland near the facial nerve necessitates careful consideration to avoid nerve injury during surgery [3-5]. In our patient, the multidisciplinary tumor board deemed systemic targeted therapy with tyrosine kinase inhibitors the most appropriate treatment option. This approach has been increasingly employed in metastatic renal cell carcinoma cases, offering improved outcomes and fewer adverse effects compared to conventional chemotherapy. The decision to opt for systemic therapy highlights the importance of personalized treatment strategies based on individual patient characteristics and tumor biology.

The rarity of parotid gland metastases from renal cell carcinoma limits the availability of large-scale clinical studies. However, the existing literature includes case reports and small case series that support our findings [1-5]. Most reported parotid metastases from renal cell carcinoma cases involve clear cell histology, consistent with our patient's presentation. The clinical course and treatment outcomes vary based on the extent of the disease, previous management of the primary renal cell carcinoma, and patient comorbidities. Despite the limited data, the literature underscores the importance of considering metastatic disease in patients with parotid swellings, especially those with a remote malignancy history.

## Conclusions

This case of metastatic clear cell renal cell carcinoma to the parotid gland highlights the significance of a comprehensive approach in diagnosing and managing rare clinical entities. The patient's history of renal cell carcinoma served as a crucial clue in considering metastasis as a potential cause of parotid swelling, underscoring the importance of meticulous history-taking in such cases. The utilization of imaging studies and biopsy facilitated the confirmation of the metastatic lesion, guiding the subsequent treatment decision. The multidisciplinary approach, involving a tumor board discussion, led to the selection of systemic targeted therapy as an effective and well-tolerated treatment option. This case emphasizes the need for a high index of suspicion for metastatic disease in patients presenting with parotid swellings, especially in the context of a previous history of malignancy. It also underscores the value of personalized treatment strategies tailored to individual patient characteristics and tumor biology in optimizing outcomes for these rare clinical scenarios.
